# Medical Communication during the Transition to Palliative Care in Pediatric Oncology in Hungary—The Parents’ Perspective

**DOI:** 10.3390/children9050651

**Published:** 2022-05-02

**Authors:** Enikő Földesi, Szilvia Zörgő, Judit Nyirő, György Péter, Gábor Ottóffy, Peter Hauser, Katalin Hegedűs

**Affiliations:** 1 Institute of Behavioural Sciences, Semmelweis University, Nagyvárad tér 4, 1089 Budapest, Hungary; zorgo.szilvia@med.semmelweis-univ.hu (S.Z.); nyiro.judit@stud.semmelweis.hu (J.N.); hegedus.katalin@med.semmelweis-univ.hu (K.H.); 2 Hemato-Oncology Unit, Heim Pál Children’s Hospital, Üllői út 86, 1089 Budapest, Hungary; peter.gyorgy@heimpalkorhaz.hu; 3 Department of Pediatrics, University of Pécs, József Attila utca 7, 7623 Pécs, Hungary; ottoffy.gabor@pte.hu; 4 2nd Department of Pediatrics, Semmelweis University, Tűzoltó utca 7–9, 1094 Budapest, Hungary; drhauserp.gyekig@bazmkorhaz.hu; 5 Velkey László Child’s Health Center, Borsod-Abaúj-Zemplén County Central Hospital and University Teaching Hospital, Szentpéteri kapu 72–76, 3526 Miskolc, Hungary

**Keywords:** child, end-of-life care, Eastern Europe, Hungary, medical communication, palliative care, parental perspective, parents, pediatric oncology

## Abstract

The transition to palliative care (PC) is a critical aspect of pediatric oncology, and it requires a high level of communication skills from doctors, which could be best judged by the parents of children who have died from cancer. Our aim was to explore the parents’ perspectives regarding the timing of the consultation on the implementation of PC, as well as facets of verbal and nonverbal communication in Hungary. Semistructured interviews were conducted with parents who had lost a child to cancer within the past 1–5 years. Interview transcripts (n = 23) were scrutinized with interpretative phenomenological analysis. The parents frequently associated palliation with end-of-life care and they clearly delimited the transition to PC after curative treatments had been exhausted. The parents were ambivalent with regard to the use of the word “death” during this consultation, and they often did not receive information on what to expect (e.g., regarding symptoms) or on who to turn to for further information or support (e.g., concerning bereavement). Although significant progress could be observed in the organization of pediatric palliative care in Hungary, there is still no widely accepted communication method for the transition to sole PC. There is a need for a culturally sensitive approach to refining the recommendations on the word use and communication protocol in pediatric PC in Hungary.

## 1. Introduction

The survival rate of children with cancer has increased markedly in the past fifty years; in high-income countries, it exceeds 80% [1]. Nevertheless, malignant diseases are still one of the leading causes of death among children and adolescents in these countries [2]. Palliative care (PC) is a critical aspect of pediatric oncology; however, the discourse around this topic has only gained momentum in the last decade. As part of this professional discourse, general international guidelines have been developed worldwide [3,4,5,6]. These guidelines emphasize the need to relieve the suffering of pediatric patients living with life-limiting diseases, and to acknowledge the importance of complex pediatric palliative care (PPC) in a multidisciplinary setting that addresses physical, psychological, and social dimensions.

Recent guidelines commonly state that PPC should begin at the diagnosis of the disease, and as palliation has long been equated with end-of-life care, the notion of the “early integration” of PPC has prompted a new set of conversations and challenges. Early integration is still not widely applied; its implementation faces a number of barriers, such as the lack of available resources and educational programs, as well as questions surrounding physician attitudes [7,8,9].

The literature that maps parental conceptions of PPC, and their attitudes in relation to its timing and implementation, is scarce. The existing studies indicate that there may be a discrepancy between parental attitudes and doctors’ perceptions of those. Some studies have found that physicians perceive increased anxiety in parents when discussing PC, while others report that the parents retained a mainly positive attitude towards PC, especially when it alleviated their child’s symptom burden [10,11]. Thus, the optimal timing of discussing PC with the parents of pediatric cancer patients within the trajectory of care is still unclear, as is whether language and word use play key roles in parents’ perceptions of PC.

With regard to palliative care, there have been significant developments in the last decade in Hungary: for the 250 newly diagnosed cases treated in the seven pediatric oncology centers in Hungary, two pediatric hospice houses were established, PPC training programs have been implemented, and pediatric palliative home care is available to an estimated 40% of the population [12]. In relation to this development, our focus in this study was on the parents’ perspectives, along the dimensions of the timing of this discussion, the preferred language, and word use in Hungary.

## 2. Methods

### 2.1. Sampling Considerations and Recruitment

To ensure that palliative referral can be realistically accomplished, we recruited parents from three oncology centers in Hungary, where a child hospice center is located within a 20 km radius. First, we approached the heads of all the hospital wards (n = 5) in the three locations to aid us in recruiting interviewees. The directors reached out to specialists who, in turn, contacted potential interviewees over the telephone (n = 24) or via regular mail (n = 10) to inquire whether they would be open to participating in the study anonymously to help doctors improve their communication skills. Three recruited parents receded from being interviewed because of either emotional distress (n = 1) or with no further explanation (n = 2), while no one answered our request via mail. Provided the parent said yes, his/her contact information was passed on to a clinical psychologist, who contacted the parent(s) and subsequently conducted the interview. The rationale for such a recruitment method was that specialists were the only ones who had direct and legal access to the parents’ contact information. The same process was repeated at all three locations from the timeframe from November 2017 to January 2020.

We included parents who had lost their child to cancer more than one but less than five years prior to our recruitment; we aimed for this timeframe so as not to infringe on the parents’ grieving processes, but so as to still be able to access relatively recent memories of these events. No parents who fit the inclusion criteria were excluded from the study.

### 2.2. Data Collection Tools

Our primary data collection tool, which was our semistructured interview guide, consisted of 18 questions and additional probes regarding the discussion(s) on the transition to PC, which can be categorized into the following themes: (1) the timing; (2) the participants; (3) the advantages/disadvantages of a psychologist being present; (4) the characteristics of the medical communication (positive/negative verbal and nonverbal facets, word use); (5) changes in the parent–child relationship after the discussion; (6) the help received concerning bereavement. This paper focuses on the timing, the participants, and the characteristics of the medical communication. We also employed a short survey at the end of each interview to record the demographic and basic clinical variables.

Interviews were audio-recorded and transcribed verbatim. Interviewees were offered optional psychological assistance following the interview.

### 2.3. Coding and Analysis

To perform free-coding and then identify the salient themes inductively, IPA (interpretative phenomenological analysis) was used. Interpretative phenomenological analysis is a qualitative research methodology that focuses on the subjective experiences of individuals. The aim of this approach is the exploration and interpretation of the individual lived experience [13].

The codes were, in part, adopted from our previous study on physician attitudes and experiences with regard to the transition to PC [14]. We also developed codes inductively for the present study in a process that involved several steps. Initially, two raters worked autonomously on the same subset of interviews. Following this, they separately created a tentative code structure that contained both the adopted codes and their inductively created ones, and they triangulated the integrated code-tree versions to create a tentative code structure. Subsequently, two raters autonomously employed this tentative code tree in another subset of interviews and triangulated their results. After this, we finalized our code tree, and two raters applied it deductively to code the entire narrative corpus by using Atlas.ti 8 (Atlas.ti Scientific Software Development GmbH) software, and by comparing the coding decisions at regular intervals during the coding process. Discrepancies were negotiated with a third researcher to produce the final coded narrative corpus. Coded fragments were analyzed according to the code by using de- and recontextualization.

Participants in the study provided informed consent to participate anonymously. Approval was obtained from the Semmelweis University Regional and Institutional Committee of Science and Research Ethics, Reference Number: SE-TUKEB 96-1/2016.

## 3. Results

### 3.1. Sample Characteristics

Semistructured interviews were conducted at Semmelweis University (n = 13), in rural clinics (n = 5), or at the interviewee’s home (n = 5). The interviews spanned from 22 to 66 min and lasted 42 min on average. Our sample consisted of 28 parents (females n = 21, males n = 7; age range: 28–55 years, mean: 38 years) who spoke to us about a total of 21 children in 23 interviews. In five cases, both parents attended the interviews; in two cases, both parents were interviewed separately. Table 1 contains the demographic and clinical details on the pediatric patients. Of the parents in our samples, 22 were married couples at the time of the interview, and 19 held university degrees. The most common pediatric malignancies are leukemias, which also have the best prognosis (over 80%); however, our sample consisted only of solid tumors, with generally worse outcomes [15]. Most of the patients (14/21) had central-nervous-system tumors. All the doctors mentioned during the interviews had worked for more than 5 years in the field of pediatric oncology as specialists.

### 3.2. Code Tree

Below, we elaborate on our results concerning the codes that relate to the timing and the language of the discussion on the transition to PC. Table 2 is a simplified version of our code tree that indicates the codes that were analyzed in the present study. The quotes from the interviewees are presented in quotation marks and italics and are cited according to the interview identifier (ID). The main focuses of the research were regarded as “high-level code” in our code tree, followed by a “mid-level” and more specified approach, and “low-level” codes, which showed more detailed content.

### 3.3. Timing of the Discussion on the Transition to Palliative Care

#### 3.3.1. Types of Timing

The parents recounted experiences that could be categorized into two types of timings: (1) at the point of diagnosis (n = 2); and (2) later in the curative treatment trajectory, or after the exhaustion of curative treatments (n = 21).

Most parents reported discussing the transition long after diagnosis, and this usually occurred in direct response to an imaging test result (e.g., MRI, CT), an unsuccessful curative treatment, or during a sudden deterioration in the child’s condition. As one parent remarked: *“[We discussed it] after the PET scan, no, actually, they waited for another round of chemo. And when we started that and didn’t get the reaction we were hoping for, then”* (ID:17).

In two cases, when the type of tumor had a less than five percent five-year survival rate, this discussion happened during the first physician–parent consult at the point of diagnosis. In one of these cases, the parent reported tension upon hearing the expression “palliative treatment” and hoped that their child *“wouldn’t get to that point”* (ID:19).

Most parents pinpointed a single conversation when curative treatments were exhausted, and they could recall the exact date of that discussion. In one case, the two parents identified different discussions when they thought that the transition to PC had been addressed, despite both of them being present at both of the mentioned discussions.

As a precursor to PC discussion, 15/23 parents claimed that, earlier in the treatment trajectory, the doctor had consulted with them at least once about the possibility that the child might die from the disease. However, in seven interviews, the parents voiced a lack of information about the possibility of their child dying: *“We didn’t communicate about it because [the doctor] also thought that the child would survive”* (ID:20). Despite not receiving this information from the physician, the parents reported that they did not have the impression that they were “unaware of the truth,” as they had considered this possibility on their own. One parent remarked, *“We knew children died of such diseases, we had heard about it. It was not told directly to us, but I knew children died of it”* (ID:15).

#### 3.3.2. Parents’ Opinions on Timing

The parents’ opinions on the timing of the discussions regarding the transition to PC were that either it was adequately timed (n = 19), that its timing was too late (n = 2), or they retained a neutral opinion (n = 2). None of our interviewees perceived the timing to be too soon.

Most parents found the timing of the discussion to be appropriate, and they frequently noted that the physicians “did everything they could” to save the child: *“The discussion was timely. All MRIs preceding it had good results, and so did the blood work. So, the timing was suitable”* (ID:7). The parents also emphasized that they appreciated hearing this information as it became relevant because it aided them in dealing with their child’s death: *“[The doctor] had to tell us, because if you keep on hoping [your child will recover], that’s not good either, I think”* (ID:4).

When the discussion was deemed too late, the parents remarked that the doctor *“should have spoken to us earlier”* because, this way, they did not have an opportunity to search for other possible therapies (ID:13).

### 3.4. Verbal and Nonverbal Communication during the Discussion

#### 3.4.1. Verbal Communication

We analyzed how the physicians formulated the information during the discussion, according to the parents. The following subcodes were distinguished: (1) “verbal positive”; and (2) “verbal negative”. The “verbal positive” contents (19/23) were divided into “verbal contents” and “features of the physician” (Table 3).

Negative verbal content, on the basis of the parental experiences, was found in eight interviews. This content included words that were judged as hurtful by the parents, or content that the parents missed from the communication, which is highly diversified (Table 4).

#### 3.4.2. Use of the Word “Death”

According to the parent recollections, the word “death” was used by the physician in 6/23 cases, and was avoided in 13/23 cases by the substitution of it with other expressions, such as: “the child will pass away”; “the disease has spread”; “the child is in a very bad condition”; and “the child does not have a chance”. In four inteviews, the parents could not recall if the word “death” was employed by the physician or not.

We also examined the parents’ opinions on the use of the word “death,” regardless of whether the doctor employed the word. There were three prevailing opinions: parents agreed with its use (n = 10), disagreed (n = 7), or could not judge (n = 4) whether it was beneficial (Table 5).

#### 3.4.3. Nonverbal Communication

The parents chiefly equated positive nonverbal communication with the physician’s ability to demonstrate empathy through his/her mannerisms and gestures. In some interviews, the nonverbal content highlighted an important aspect of PC consultation, which is its location, which the physician chose. Negative manifestations of nonverbal communication were described in only two interviews, with both referring to the milieu and to the functioning of the ward (Table 6).

## 4. Discussion

In our previous qualitative study, which involved more than 70% of the physicians who work in PPC, we found that many of the physicians associated palliative care with end-of-life care, and that they only introduced palliation after exhausting the curative treatments in Hungary [14]. Although a desire for multidisciplinary teamwork could be observed, the physician’s role corresponded with the “Solo Practice Model”, which is described by Bruera, in which the oncologist attempts to take care of all patient-related issues [14,16].

Our present study scrutinized the consultation(s) about the transition to PC, during which the physician informs the parents of a child undergoing treatment for a malignancy, that there are no more curative options left, and that they can only provide palliative care from this point onwards.

### 4.1. Timing of the Discussion on the Transition to Palliative Care

In conveying information about the exhaustion of curative treatments and the child’s impending death, some studies found that physicians were prone to employ indirect speech and to only address these subjects when the parents explicitly asked them. Wolfe et al. found that there may be a significant delay between the physician and the parental recognition of the child’s approaching death [17]. Perceived expectations and fears, coupled with poor communication, may thus lead to an impeded information exchange and to delays in various aspects of the PPC.

Most parents stated that this transition to PC only took place after all possible curative treatments failed. The parents’ strong delineation between the curative and palliative phases can be connected to the preconception of Hungarian pediatric oncologists that PC should only be employed after the curative options have been exhausted. This discrepancy between the local practice and the international guidelines’ recommendations on the early integration of PC may exist because of a lack of palliative professionals as well. This need has also been documented with regard to adult PC in several geographical locations [18,19]. Another explanation for the lack of early integration may be inadequate cooperation between professionals, which is further exacerbated by the fact that the curative care that is provided in hospitals is often sharply separated in space from pediatric palliative and hospice services. For two of the three hospitals in our study, the closest palliative facility was located 15 km from the pediatric oncology center, in a neighboring village. In the case of the third hospital, the palliative facility was located very close to the oncology center; the parents reported markedly good experiences with regard to this particular hospital, which can be explained by the close collaboration between the two forms of care. It is important for such institutions to work more closely together, and one possible facilitating factor can be physical proximity.

With regard to the timing of the transition to PC, studies show that physicians may be reluctant to introduce PC early because they believe it places a heavy burden on parents, contributes to their anxiety, upsets young patients and their families, and, consequently, can be detrimental to the parent–physician relationship [11,20]. The introduction of PC is regarded as a stressor; this was a reason for deferring PC consultations for physicians [21,22]. Another reason for a delay in having these discussions and for delayed PC integration is the cultural taboo of death and dying [23]. The literature generally shows stronger early integration practices in relation to high-risk cases [11,20]. Correspondingly, our findings also show that PC was introduced at diagnoses in only two cases: when poor prognoses made the physicians confident of unfavorable outcomes. Furthermore, parents may disagree on when the consultation on the transition to PC actually took place, as was the case with two parents in our study. Bluebond-Langner reported similar findings: the parents of children with high-risk brain tumors, and who were present at the same consultations, interpreted the prognostic information differently [24].

The strong associative relationship between death and palliation is evident when parents suggest renaming the expression by changing it from “palliative care” to “supportive care” [7,11]. This may be a prevalent opinion among physicians also, as one Canadian study found that pediatric oncologists refer patients earlier if the PC is termed as “supportive care” [7].

There has been a significant improvement in the timing of PPC consultations worldwide: in 2009, a median of merely 8 days elapsed between the PPC consultation and the child’s death; however, by 2015, this time period increased to 1.6 months, which leaves a 17.2-month median from primary diagnosis to the first PPC consultation [25,26]. Although this time interval was not checked, almost all of the parents (19/23) considered the timing of the transition to PC to be appropriate, and nobody deemed the discussion too early. This may be due to a particular coping method: as long as curative treatments are still available, it cultivates hope in parents, of which they can hold on to and draw strength from. Our study confirmed this when the parents voiced that, as long as the child was in good condition, they saw no point in mentioning PC, even if the disease was incurable. Congruently, the parents in our study who considered the transition to PC to be too late felt that hope was taken away from them through their inability to try other treatments abroad. In contrast, by recording the consultations between physicians and parents, and by conducting interviews with 28 parents, Nyborn et al. concluded that, although most of the parents found the prognostic information upsetting, the majority also valued honest communication about the prognosis. The parents noted that frank disclosure fostered hope by relieving uncertainty, and by allowing them to make the best possible decisions for their children [27].

### 4.2. Verbal and Nonverbal Communication during the Discussion

From the physician’s viewpoint, studies show that conveying empathy, honesty, hope, and detailed information while avoiding medical jargon were important considerations [28]. Hsiao et al. identified five domains of physician communication that are deemed highly salient and influential in the quality of care. These include: relationship building; the demonstration of effort and competence; information exchange; availability; and the appropriate level of child and parent involvement. The parents identified the coordination of care as another important communication domain [29]. Hentea et al. determined four major themes that parents recognized as important: the communication style, the content, the logistics, and the healthcare team [30]. In our cohort, the parents were able to recall a wide range of positive verbal content, which reflects the fact that the physicians in our study generally used good communication techniques in the conversations, keeping in mind the primacy of building a partnership with the parents rather than considering them inferior in any way. Waldman et al. describe five main domains that mirror the areas that are typically covered in an initial PPC consultation and that focus on exploring the goals of care: “Who is your child?”; understanding the illness and the prognosis; hope; worries and fears; and supports [31]. These elements also occurred in the reflections of the parents in our study. The physicians’ choices of words expressed support and empathy, which ensures that the child’s needs and interests are in focus. The negative verbal content mainly included the lack of information about the period after the transition to PC. The close control that families are used to during curative treatments was replaced by uncertainty and a lack of knowledge. The parents had difficulties finding anchors in such a situation; thus, they experienced loneliness, helplessness, and incompetence.

Research has found individual and cultural differences that surround specific phrases (e.g., “cancer”), and a preference for direct or indirect language with regard to the child’s dying process and death [32,33]. The word “death” may appear to have a harsh official tone in Hungarian, and it is commonly used to express something factual; hence, for many, it may not be considered part of empathetic communication [23]. Despite ambivalent attitudes in several cultures toward the use of this word, the protocols generally suggest employing clear language that explicitly employs the word “death” in order to make the situation unequivocal to parents [34]. In an earlier study, we found that only 26% of the physicians used this word, despite international protocols and the communication training that all medical students receive in Hungary [23]. This may be due to their own fear, the perceived expectations of the parents, the physicians’ own ambivalent feelings toward being explicit, or their attitudes regarding death. The parents in our study were also ambivalent: 43% agreed with employing this word, 30% disagreed with it, and 17% could not decide.

The parents in our study emphasized physician communication through nonverbal channels as well. Among the actions that were interpreted as positive were: (1) the physical expression of attention (through posture and gestures such as eye contact and nodding); and (2) conveying support in this emotionally taxing situation (through gestures that express empathy and understanding). Some of the parents also pointed out that creating the appropriate conditions for the discussion (e.g., location, quiet, switching off mobiles) sends a positive message. The actions that were interpreted as negative were connected to failing to secure intimate surroundings for the discussion and failing to switch off their phones. Overall, the verbal and nonverbal communication of the physicians was considered to be appropriate by the parents; they reported a sense of partnership with the attending physician.

Our study had several limitations. The interviewees were not selected systematically but were chosen at the treating physicians’ discretion, who were presumably more likely to reach out to parents with whom they had fostered a good relationship. Our research questions and design did not prioritize conducting the interviews immediately after the discussion regarding the transition to PC or securing a recording of the actual consultation. We were mindful of the parents’ grieving processes and privacy. Therefore, as with all instances of recall, the parents’ memories were subject to time and to post hoc modifications, and without recording the actual consultation, certain details about it were inevitably lost. Moreover, our small sample size lends insight into the attitudes of the parents in our cohort, but the level of transferability to Hungarian parents, in general, is unclear, and much less so for other geographical areas and cultures. On the other hand, it is our conviction that our findings provide a supplement to the exploration of parental perspectives toward PPC within a specific sociocultural environment.

## 5. Conclusions

As vital stakeholders in PPC, the parents’ preferences, needs, and attitudes should be considered when refining clinically informed recommendations and protocols for PPC. With regard to PC, overall, both physicians and parents seem to have lingering associations with end-of-life care in Hungary, which is a crucial barrier to the early integration of PC, and which impedes discussions with regard to palliation in general.

Furthermore, albeit recommendations have urged the explicit use of the word “death”, both physicians and parents remain ambivalent with regard to this in Hungary. On the basis of our findings and of others from culturally diverse populations, the univocal use of the word “death” may be worth reconsidering, as recommendations are generally based on Western cultural norms and may not reflect non-Western perspectives.

## Figures and Tables

**Table 1:** Demographic and clinical characteristics of patients (n = 21). List of abbreviations: MBL: medulloblastoma; GBM: glioblastoma; NBL: neuroblastoma; RMS: rhabdomyosarcoma; cPNET: central primitive neuroectodermal tumor; EPM: ependymoma; ATRT: atypical teratoid rhabdoid tumor; GCT: germ cell tumor; ES: Ewing sarcoma; NC: NUT midline carcinoma.

Category	Feature
Histological diagnosis	MBL (4), GBM (3), NBL (3), RMS (3), cPNET (2), EPM (2), ATRT (1), GCT (1), ES (1), NC (1)
Sex	Females (9); Males (12)
Age at time of death	Median: 5.5 years (9 months–19 years)
Time between diagnosis and death	Median: 23 months (3 months–8 years)
Location of treatment center/ Number of patients	Semmelweis University, Budapest (12) Heim Pál Children’s Hospital, Budapest (3) University of Pécs, Pécs (6)

**Table 2:** Simplified code tree; coding was performed with low-level codes.

High-Level Code	Mid-Level Code	Low-Level Code
1. Process	1.1 Timing	1.1.1. At diagnosis
		1.1.2. During/end of curative treatment
		1.1.3. Other
	1.2 Parent’s opinion on timing	1.2.1 Too early
		1.2.2 Too late
		1.2.3 Appropriate
		1.2.4 Other
	1.3 Mention of possible death	1.3.1 Yes
		1.3.2 No
		1.3.3 Does not remember
2. Language	2.1 Verbal communication	2.1.1 Positive
		2.1.2 Negative
	2.2 Nonverbal communication	2.2.1 Positive
		2.2.2 Negative
	2.3 Use of the word “death” during discussion	2.3.1 Yes
		2.3.2 No
		2.3.3 Does not remember
	2.4 Parent’s opinion on using the word “death”	2.4.1 Agrees
		2.4.2 Disagrees

**Table 3:** Categories, examples, and quotations of Code 2.1.1 (positive verbal communication).

Category	Example	Quotations
Content	Sustainment of hope	*“It was so good telling us that hope never dies.”* (ID: 20)
	Experiences together with the child	*“[…] try to continue normal life and collect as much experiences as possible, together.”* (ID: 2)
	Lack of pain and suffering	*“They take care not to have the child pain.”*
	Parents could try other opportunities	*“[…], but he did not pull back us to try other ways.”* (ID: 15)
	The child could be brought home	*“Yes, that we could bring the child home.”* (ID: 5)
	Spirituality, waiting for a miracle	*“When he mentioned these spiritual things, like eternity and love connections, these words were the key words that day.”* (ID:19)
	They can receive practical support/Hospice care is available	*“It was emphasized a lot, that they wanted to know, how we will manage this situation.”* (ID: 12)
	Relief of the responsibility	*“When he said, they offered all possible treatments to child. So, they have relieved all of us of responsibility”* (ID: 8)
	Information about the child’s experiences	*“[…] we go home and it seems, he is playing lego or doing something else, but he can read on our face, on our body language and on our whisper while adults are talking, or making a phone call, when we think, he does not listen to us.”* (ID: 16)
Features	To be honest	*“They should be honest, even if it hurts.”* (ID: 21)
	To be patient	*“He explained us very nicely and with patience, how it worked, what were the consequenses and he explained us, what we had not understood in those things before.”* (ID: 16)

**Table 4:** Categories, examples, and quotations of Code 2.1.2 (negative verbal communication).

Category	Example	Quotation
Word	“mummy” (a special Hungarian word for “mother” with diminutive)	(*“What I can call as hurtful is “mummy”* (ID: 6)
	Healing/Cure	*(“[…] Question: He will stay alive, but is he not cured? Answer: Yes, but he lives with a disease or disability.”* (ID: 1)
Lack of information	Possible period of time until death of the child	*“Because if they tell 3–6 months, what does the parent hear? 6 months and it’s correct so. […] we were very disappointed because we planned to show him this and that and we could take him nowhere because of the big oxigene bottles and of his condition…”* (ID: 5)
	They did not speak about hospice	*“hospice-house as an opportunity was not discussed.”* (ID: 16)
	If they go home with the child, what could be expected during the nursing, and what should they be prepared for?	*“Maybe if a little bit about this medical part,* e.g., *when should we come back to hospital or what we should see or not see, have been discussed, this could have been helpful”* (ID: 5)
Other	Physicians on the ward communicated different contents	*“The treating physician told us that our son was incurable […] the other physician told us later, it was too early to declare that our son would die.”* (ID: 8)
	Empty promises and the taboo that the child could die	*“Father: That was a taboo the whole time, not the hospice-house itself, but this opportunity. Mother: That the child could die, too.”* (ID: 6)

**Table 5:** Categories and quotations of Code 2.4 (parents’ opinions on the use of the word “death”: agree: 2.4.1; disagree: 2.4.2; could not judge: 2.4.3).

Category	Quotation
Parents agree (n = 10)	*“I think it can be used. I think the context is important; in a proper, not unfriendly context it could be said by the physician”* (ID:18)
Parents disagree (n = 7)	“*This is not good, I think. It scares me*” (ID: 13).
Parents could not judge (n = 4)	“*I would tell the poor doctor who is in that situation that they should assess whether the parent needs them to use this word or not”* (ID:3).

**Table 6:** Categories, examples, and quotations of Code 2.2 (nonverbal communication: positive: 2.2.1; and negative: 2.2.2).

Category	Example	Quotation
Nonverbal positive	Demonstrates empathy	*“Talking very calmly, he told us what happened, point by point, and radiated warmth and friendship—or I don’t know how to describe it—and we could see he was empathetic, he was very sorry to tell us these things”* (ID:21).
	Location and circumstances of PC consultation	*“[This discussion] should not be in the hall or the canteen […] The parent should really feel that this is an intimate situation and the doctor sacrifices his time and attention, and waits for the parent to react….”* (ID: 16)
Nonverbal negative	Body language of the staff	*“It would have helped a lot, if the atmosphere of the ward was good and more empathic”* (ID: 1)
	An unwelcome participant witnessing this conversation	*“[This nurse] always disturbed me. She was always sitting there in the next room with the door open”* (ID: 6)

## Data Availability

The datasets used and/or analyzed during the current study are available from the corresponding author upon reasonable request.
